# Type A Aortic Intramural Hematoma Without Identifiable Intimal Tear Presenting With Minimal Initial Findings

**DOI:** 10.7759/cureus.108743

**Published:** 2026-05-12

**Authors:** Leon Popaj, Keith Cronovich

**Affiliations:** 1 Department of Emergency Medicine, Henry Ford Health System, Clinton Township, USA

**Keywords:** acute aortic syndrome, aortic intramural hematoma, aortic media hemorrhage, ascending aortic pathology, intramural hematoma, non-dissection aortic disease, type a intramural hematoma

## Abstract

Aortic intramural hematoma (IMH) is a subtype of acute aortic syndrome characterized by hemorrhage within the aortic media in the absence of an identifiable intimal tear. Although IMH represents a less frequent presentation of acute aortic syndrome, involvement of the ascending aorta (type A) carries significant morbidity and mortality comparable to classic aortic dissection. Type A IMH has been reported to progress to acute aortic dissection and is associated with an increased risk of aortic rupture due to thinning of the outer aortic media.

We report a 77-year-old female with hypertension presenting with acute-onset substernal chest pain and minimal initial findings, including a non-ischemic electrocardiogram and negative serial troponins. Chest radiography demonstrated a widened mediastinum initially attributed to projectional artifacts. Computed tomography angiography (CTA) subsequently revealed a type A intramural hematoma extending from the ascending into the descending thoracic aorta without a visible intimal flap.

This case highlights the diagnostic challenge of IMH in hemodynamically stable patients and underscores the importance of maintaining a high index of suspicion and early advanced imaging in suspected acute aortic syndromes.

## Introduction

Acute aortic syndromes (AAS) represent a spectrum of life-threatening aortic pathologies that include classic aortic dissection, intramural hematoma (IMH), and penetrating atherosclerotic ulcer. IMH accounts for approximately 5-20% of AAS and is defined by hemorrhage within the aortic media without a visible intimal tear on imaging [1,2].

IMH is now recognized to exist along the same pathophysiologic continuum as classic dissection, despite the fact that it was once thought to be different. Significant morbidity and mortality are linked to type A IMH, which affects the ascending aorta; in-hospital mortality rates in untreated patients have been reported to exceed 20-40% [2]. If not identified and treated right once, the condition may progress to overt dissection, rupture, or cardiac tamponade.

Because patients frequently present with nonspecific symptoms and may demonstrate reassuring initial laboratory and electrocardiographic findings, diagnosis can be delayed. We present a case of type A IMH identified in the emergency department despite minimal initial findings, emphasizing the importance of clinical suspicion and early advanced imaging.

## Case presentation

A 77-year-old female with a history of hypertension, radiculopathy, and gastroesophageal reflux disease presented to the emergency department with acute-onset substernal chest pressure that woke her up from her sleep. She described the pain as severe and sudden in onset, stating it felt like “an elephant sitting on her chest.” She denied prior similar episodes.

Aspirin 324 mg and nitroglycerin were administered as part of prehospital care, which reduced discomfort from 10/10 to 6/10. Her blood pressure was 127/73 mmHg, her heart rate was 60 beats per minute, her breathing rate was 16 breaths per minute, her oxygen saturation was 96% on room air, and she was afebrile when she arrived.

Electrocardiography demonstrated sinus bradycardia with premature atrial complexes and no ischemic changes. Serial troponins remained within normal limits. Laboratory studies were notable for mild leukocytosis (WBC = 12.1-13.0 ×10³/µL), mildly elevated B-type natriuretic peptide (194 pg/mL), and an estimated glomerular filtration rate of 58 mL/min/1.73 m² (Table 1).

**Table 1:** Initial laboratory findings on presentation. Initial laboratory evaluation demonstrated mild leukocytosis, mildly elevated B-type natriuretic peptide levels, normal troponin levels, and mildly reduced renal function as evidenced by a decreased estimated glomerular filtration rate. These findings were nonspecific but contributed to the overall clinical assessment during evaluation for acute aortic pathology.

Laboratory test	Patient value	Reference range	Interpretation
White blood cell count (WBC)	12.1-13.0 ×10³/µL	4.0-10.5 ×10³/µL	Mild leukocytosis
B-type natriuretic peptide (BNP)	194 pg/mL	<100 pg/mL	Mildly elevated
Troponin I	6 ng/L	<19 ng/L	Normal
Estimated glomerular filtration rate (eGFR)	58 mL/min/1.73 m²	≥90 mL/min/1.73 m²	Mildly reduced renal function

**Figure 1:**
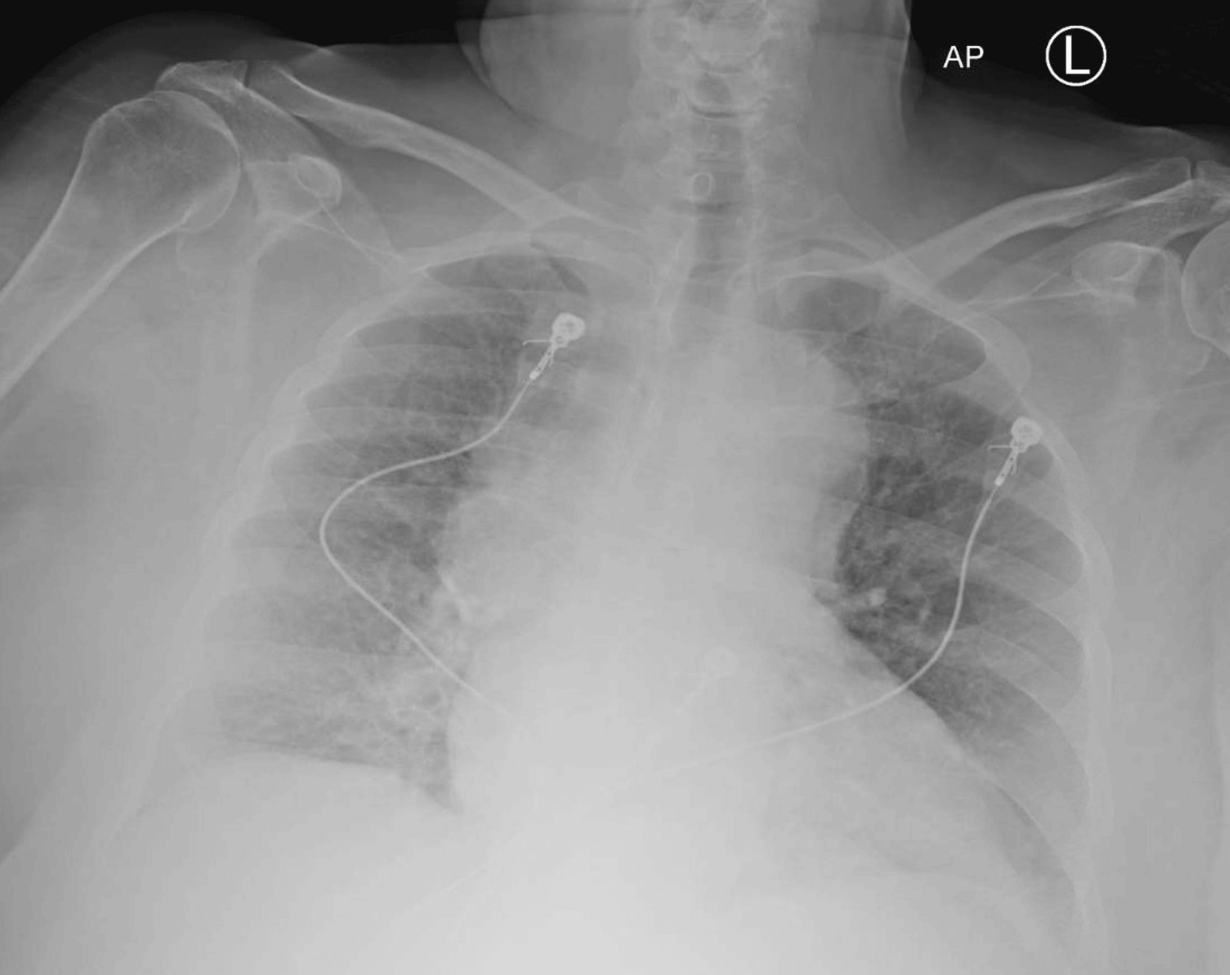
Anteroposterior chest radiograph demonstrating apparent mediastinal widening. Initial chest radiography demonstrated apparent mediastinal widening on portable anteroposterior imaging, prompting concern for possible acute aortic pathology and subsequent computed tomography angiography evaluation.

Chest radiography demonstrated a widened mediastinum, initially favored to represent projectional artifacts due to the anteroposterior technique (Figure 1). However, given the patient’s advanced age, abrupt symptom onset, and persistent clinical concern for acute aortic pathology, computed tomography angiography (CTA) of the chest, abdomen, and pelvis was obtained.

**Figure 2:**
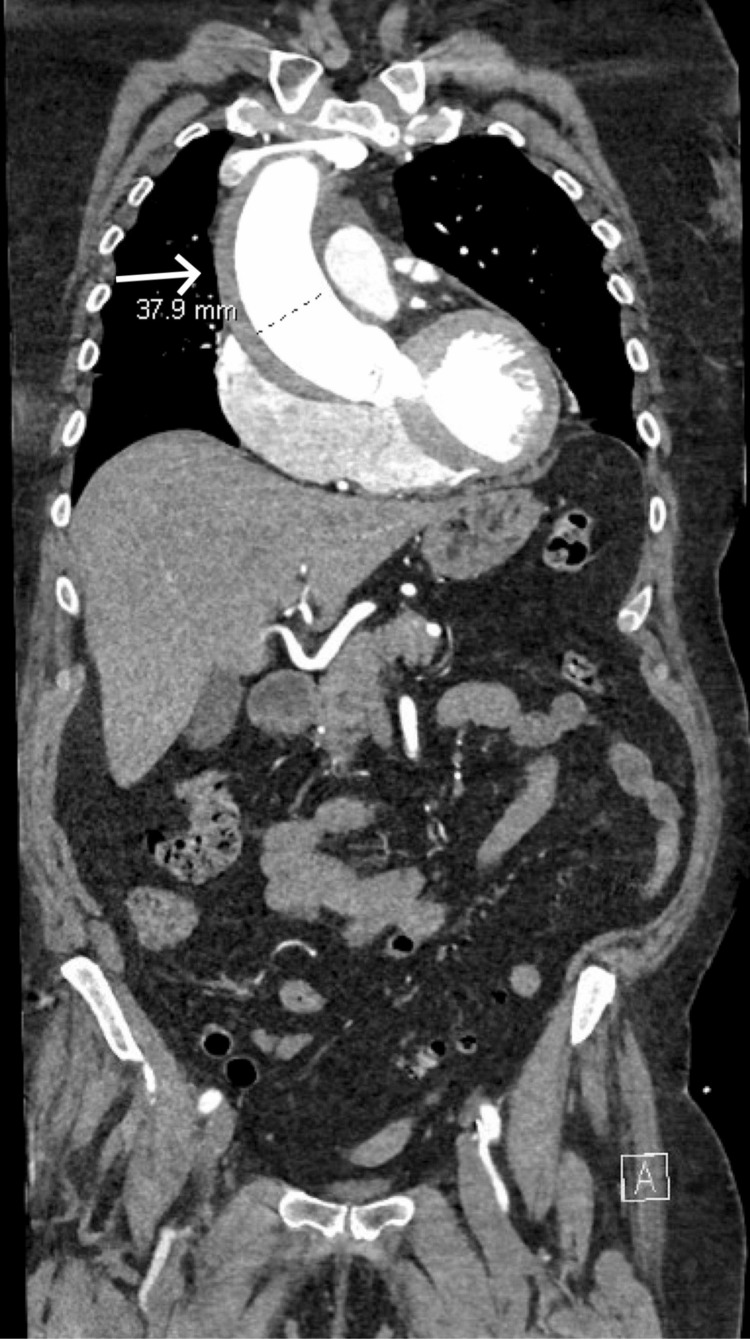
Coronal computed tomography angiography (CTA) demonstrating type A aortic intramural hematoma. Coronal CTA shows crescentic, hyperattenuating thickening of the ascending aortic wall (arrow), measuring approximately 37.9 mm, consistent with intramural hematoma. The abnormality extends from the sinotubular junction into the descending thoracic aorta. Notably, there is no visible intimal flap or double lumen, distinguishing this from classic aortic dissection.

CTA revealed crescentic thickening of the ascending aortic wall extending from the sinotubular junction through the descending thoracic aorta, terminating near the aortic hiatus prior to the celiac artery origin (Figure 2). These findings are characteristic of intramural hematoma, which classically appears on CTA as crescentic or circumferential aortic wall thickening without contrast enhancement or an identifiable intimal flap [3].

**Figure 3:**
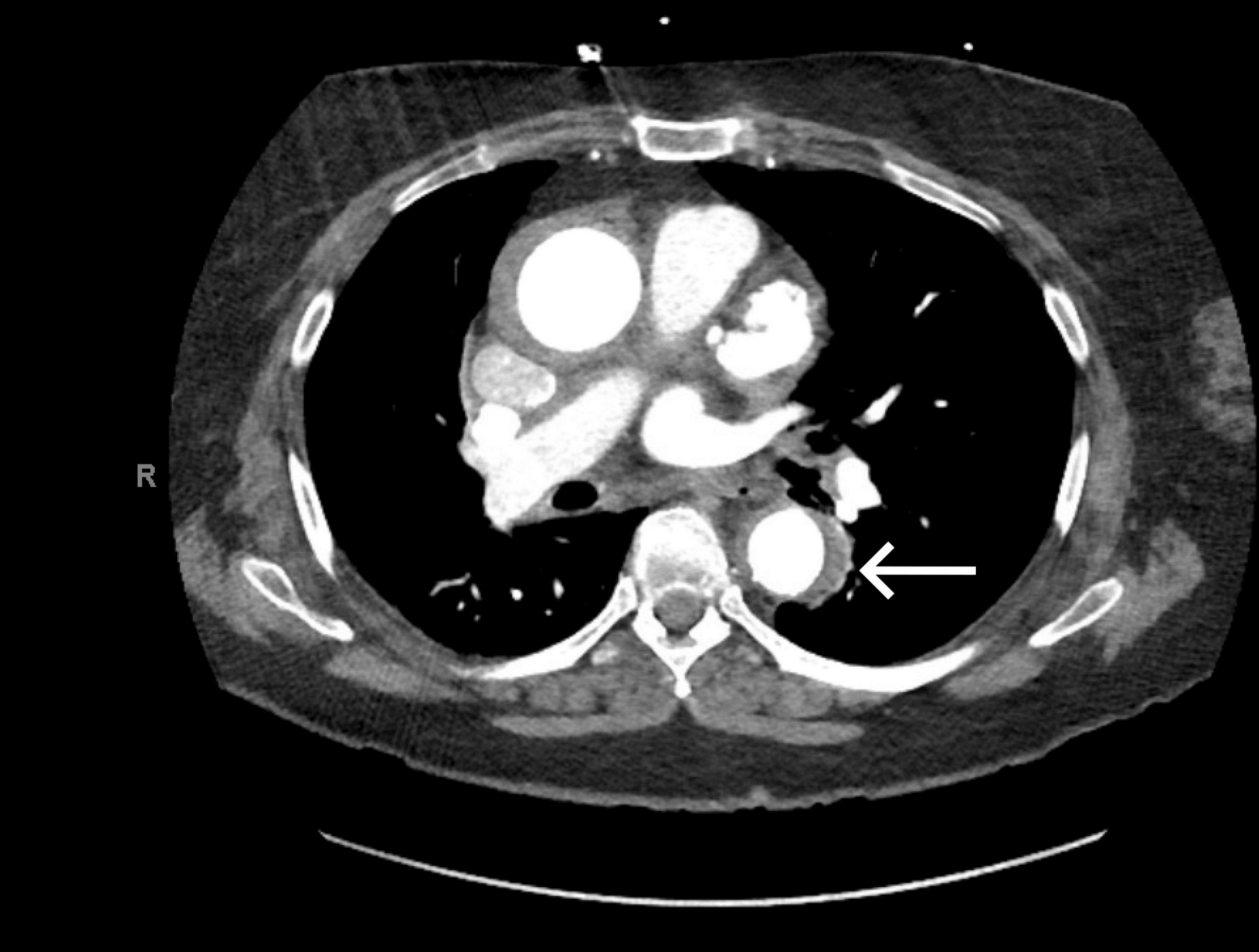
Axial computed tomography angiography (CTA) demonstrating absence of intimal flap. Axial CTA of the thorax demonstrates circumferential aortic wall thickening without evidence of an intimal flap or separation into true and false lumens. The absence of contrast within the thickened wall supports the diagnosis of intramural hematoma rather than classic aortic dissection.

Axial imaging further demonstrated circumferential aortic wall thickening without evidence of an intimal flap or separation into true and false lumens (Figure 3). The absence of an intimal flap is a key distinguishing feature separating intramural hematoma from classic aortic dissection [2,3].

There was suspected involvement of the brachiocephalic artery and proximal right common carotid artery. No double lumen was identified, supporting the diagnosis of type A intramural hematoma rather than classic dissection.

Cardiothoracic surgery was emergently consulted. The patient was initiated on intravenous esmolol infusion at 50 mcg/kg/min and nicardipine infusion at 5 mg/hr for anti-impulse therapy, with titration to target heart rate and blood pressure goals, including a heart rate of less than 60 beats/min and systolic blood pressure between 100 and 120 mmHg.

## Discussion

IMH was first described by Krukenberg in 1920 during postmortem examination as a “dissection without intimal tear,” a definition that later became widely accepted as the classic description of IMH [1]. IMH occurs when bleeding develops within the aortic media, most often due to spontaneous rupture of the vasa vasorum [1]. On CTA, it typically appears as crescent-shaped or circumferential thickening of the aortic wall without contrast enhancement and without a clearly identifiable intimal flap [3].

It is important to differentiate IMH from classic aortic dissection. In dissection, a tear in the intima creates separate true and false lumens, divided by an intimal flap. IMH lacks this feature, although patients can present in a very similar way, often with sudden, severe chest pain. Findings such as pulse deficits or evidence of malperfusion are less frequent but may still be present [2].

Type A IMH is associated with significant risk. Data from the International Registry of Acute Aortic Dissection (IRAD) and other studies show that, if left untreated, mortality is comparable to that of classic dissection [2,4,5]. For this reason, current American College of Cardiology/American Heart Association (ACC/AHA) guidelines, which serve as the primary evidence-based standards for cardiovascular care in the United States and classify recommendations based on strength and quality of evidence, recommend urgent surgical evaluation in patients with type A IMH [6,7]. Prompt recognition is key, as delays can allow progression to dissection, rupture, or cardiac tamponade.

From an emergency medicine standpoint, this case underscores how easily these patients can be missed. Initial testing may appear reassuring, with normal troponins and non-specific ECG findings. Even temporary symptom relief with nitroglycerin can shift concern toward a cardiac ischemic cause. Relying too heavily on these early findings risks premature diagnostic closure.

In patients with concerning features such as sudden-onset chest pain, advanced age, or a widened mediastinum, early CTA should remain a strong consideration even when the initial workup appears reassuring. This is consistent with National Institute for Health and Care Excellence (NICE) recommendations supporting early advanced imaging in selected patients presenting with new-onset chest pain when serious underlying pathology is suspected [8].

## Conclusions

Type A IMH is a life-threatening variant of acute aortic syndrome that may present with subtle findings and reassuring initial testing, making diagnosis challenging in otherwise hemodynamically stable patients. Emergency physicians should maintain a high index of suspicion and pursue advanced imaging when clinical concern persists. Early recognition, aggressive medical management, and rapid cardiothoracic surgical consultation are essential to improving outcomes. Although acute type A IMH carries morbidity and mortality comparable to classic aortic dissection, studies suggest that stable patients may benefit from timely surgical intervention within the first 72 hours following initial medical stabilization, while unstable patients often require emergent operative management. A multidisciplinary approach with individualized risk stratification remains critical for optimal care.
